# Reciprocal immune benefit based on complementary production of antibiotics by the leech *Hirudo verbana* and its gut symbiont *Aeromonas veronii*

**DOI:** 10.1038/srep17498

**Published:** 2015-12-04

**Authors:** Aurélie Tasiemski, François Massol, Virginie Cuvillier-Hot, Céline Boidin-Wichlacz, Emmanuel Roger, Franck Rodet, Isabelle Fournier, Frédéric Thomas, Michel Salzet

**Affiliations:** 1 Univ. Lille, Unité Evolution, Ecologie et Paléontologie (EEP), CNRS UMR 8198, F-59 000 Lille, France; 2 Univ. Lille, Unité Protéomique, Réponse Inflammatoire, Spectrométrie de Masse (PRISM), INSERM U 1192, F-59 000 Lille, France; 3 Univ. Lille, Centre d’infections et d’immunité de Lille, CNRS UMR 8204, INSERM U 1019, F-59 000 Lille, France; 4 MIVEGEC, UMR IRD/CNRS/UM5290, 911 Avenue Agropolis, BP 64501, 34394 Montpellier Cedex 5, France

## Abstract

The medicinal leech has established a long-term mutualistic association with *Aeromonas veronii,* a versatile bacterium which can also display free-living waterborne and fish- or human-pathogenic lifestyles. Here, we investigated the role of antibiotics in the dynamics of interaction between the leech and its gut symbiont *Aeromonas*. By combining biochemical and molecular approaches, we isolated and identified for the first time the antimicrobial peptides (AMPs) produced by the leech digestive tract and by its symbiont *Aeromonas*. Immunohistochemistry data and PCR analyses evidenced that leech AMP genes are induced in the gut epithelial cells when *Aeromonas* load is low (starved animals), while repressed when *Aeromonas* abundance is the highest (post blood feeding). The asynchronous production of AMPs by both partners suggests that these antibiotic substances (i) provide them with reciprocal protection against invasive bacteria and (ii) contribute to the unusual simplicity of the gut microflora of the leech. This immune benefit substantially reinforces the evidence of an evolutionarily stable association between *H. verbana* and *A. veronii*. Altogether these data may provide insights into the processes making the association with an *Aeromonas* species in the digestive tract either deleterious or beneficial.

Many, if not most, eukaryotic organisms have evolved intimate associations with symbiotic microorganisms^1^. Mutualistic symbiotic interactions provide multiple advantages ranging from nutritional contribution to mediation of defense against pathogens and other natural enemies^2^. An important question regarding such mutualistic interactions is to assess how such interactions can remain evolutionarily stable, with no partner evolving towards “cheater” strategies^3^,^4^,^5^. Because of the potential for external bacteria to colonize the digestive tract, the study of gut microbiota symbiosis is particularly well suited to empirically assess the functional aspects of stable mutualistic interactions as well as the importance of vertical *vs.* horizontal modes of symbiont transmission.

One way to tease apart these interspecific interactions is to use a biological model housing a simple microflora such as the medicinal leech *Hirudo verbana*. Indeed the gut of this hematophagous annelid is mostly dominated (>95%) by two Gamma proteobacteria species, *Aeromonas veronii* and *Mucinivorans hirudinis* (formerly known as *Rikenella*), the spatial and temporal population dynamics of which is well described within the host during blood digestion^6^,^7^,^8^. In comparison, the mammalian gastrointestinal microbiome contains between 500 to 10 000 distinct species or strains^9^,^10^ (Human Microbiome Project). In the leech, the two major symbionts have been proposed to enhance fitness by helping in the digestion of the blood and by providing the leech with nutrients not obtained from the diet^11^,^12^. *A. veronii* is a pioneer species in the gut of *H. verbana* and influences the recruitment of later colonizing *M. hirudinis* symbionts^13^. It is of particular interest that the medicinal leech has established a long-term association with *A. veronii*, a versatile bacterium which possesses alternative lifestyles beyond that of mutualism (free living waterborne, pathogenic for fishes and human^11^,^14^,^15^), unlike *M. hirudinis,* only described in the normal flora of intestinal tracts of mammals and birds so far^16^,^17^. A horizontal transmission mode of *Aeromonas* between leeches, complementing maternal transmission, was recently suggested, thus providing *Hirudo* with a pool of symbionts with high genetic variability to quickly adapt to environmental changes^18^.

Several factors contributing to the colonization success of the gut by the symbionts and to the unusual simplicity of the leech microbiota have been identified (for review see^19^,^20^,^21^). For example, *Aeromonas* are resistant to the immune complement factors still present and bactericidal (for few hours after meal) in the ingested vertebrate blood, thus giving them a competitive advantage over other bacteria in their ability to colonize the leech^12^. *A. veronii* and *M. hirudinis* symbionts have also developed strategies to avoid the cellular immune system of the leech. *A. veronii* uses a classic virulence factor, *i.e.* the type III secretion system (T3SS), to get protected from phagocytosis by the leech immune cells that patrol the gut^22^. *M. hirudinis* does not possess a T3SS but the polysaccharide matrix abundantly surrounding its microcolonies constitutes a barrier against phagocytosis by the host cells^6^.

In the gut, it is now well established that the immune system of the host plays an important role not only in fighting pathogenic bacteria but also in selecting and keeping the microflora under control^13^,^14^,^23^,^24^,^25^,^26^,^27^. In both invertebrates and vertebrates, this process involves antimicrobial peptides (AMPs), defined as natural antibiotics with a varying number (from five to over a hundred) of amino acids, produced by nearly all organisms^28^. Quite remarkably, it has also been shown that symbiotic bacteria participate to the immune response of the digestive tract^29^. One possible interpretation for such a behavior would be as an effort to protect their niche from other bacterial strains competing for resources in the gut. Resident commensal microorganisms indeed produce AMPs that act as anti-competitors against other bacteria, including pathogenic ones, thus indirectly benefiting the host health^30^,^31^. In this context, understanding the functioning of host-symbiont interactions requires unraveling the chemical warfare between symbiotic and pathogenic microorganisms, as well as the dissection of how the host immune response has evolved to distinguish between bacteria with different level of context-dependent pathogenicity.

In the medicinal leech, the role of the symbionts in the protection of the gut against colonization by pathogens was hypothesized early in 1953, but the immune benefits of the association have never been demonstrated^32^. More recently, Indergand and collaborators have shown that pathogenic bacteria are unable to colonize the leech gut, thus suggesting that the community is tightly controlled by the conditions created by the leech and/or the symbionts inside the gut^12^. We know that leeches produce various AMPs inducible upon bacterial infection. In previous studies, two AMPs named theromyzin (TMZ) and theromacin (TMC) were detected in the intestinal epithelium of the distantly related leech, *Theromyzon tessulatum*^33^. Later on, two orthologs of TMZ and TMC, plus two other AMPs called lumbricin (LUMB) and neuromacin (NMC) were respectively identified in the blood and in the nerve cord of the medicinal leech^34^,^35^,^36^. However, the nature of antibiotic molecules synthesized by the digestive epithelium of *H. verbana*, as well as those produced by its pioneer symbiont *Aeromonas*, has never been reported so far.

The present work aims at answering the two following questions: (i) Has a beneficial immune balance evolved between the leech and its gut microbiome? (ii) Are antibiotics involved in this interspecific interaction? First, we determine whether the gut of *H. verbana* and its pioneer symbiotic bacteria (*A. veronii*) produce antibiotics and characterize them. Second, we explore the temporal dynamics of antibiotics production, after the blood meal, when the symbiont load is high, and between meals when the symbionts become almost undetectable^6^.

## Results

### Identification of the gut symbiont

**Figure 1:**
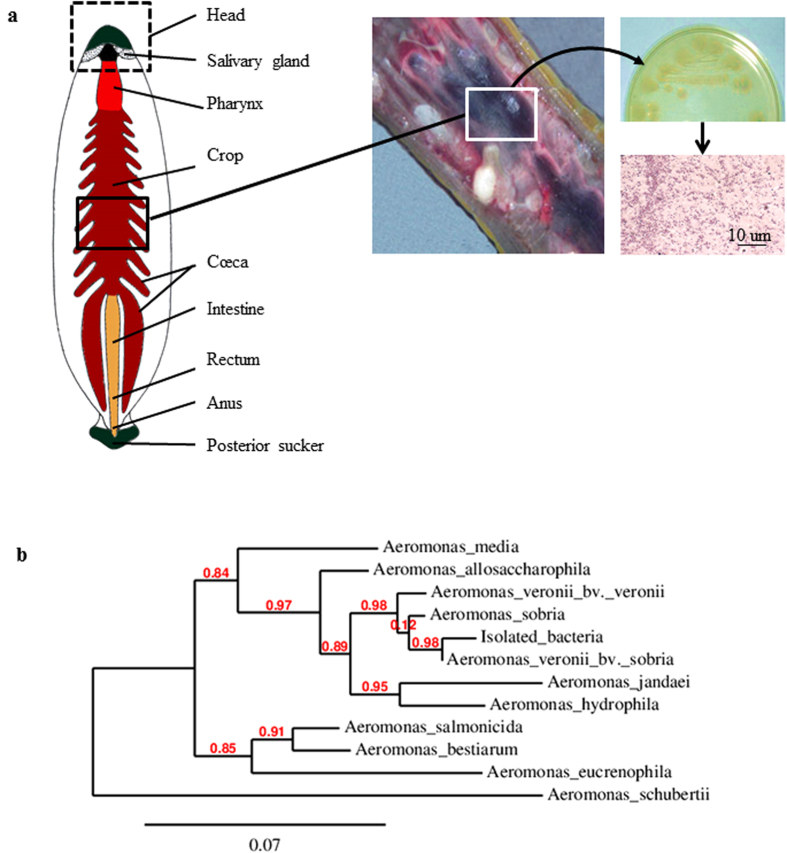
Isolation and identification of the *Aeromonas* strain present in the crop cecum of *Hirudo verbana.* The leech digestive tract is divided into two main compartments: the crop where the ingested blood is stored and the intestine where the blood digestion occurs. **(a**) The digested blood was spread on an agar plate. Colonies appear resistant to ampicillin, make the culture medium turn green and stain pink/red after a Gram-staining protocol, three phenotypic characteristics of the bacteria belonging to the *Aeromonas* genus. (**b**) Phylogenic analysis of the sequence obtained from the PCR amplification of the isolated bacteria with the primers gyrB3F and gyrB14R. The sequence presents 99% of identity with the sequence of *Aeromonas veronii bv sobria* (AY101791). The phylogenic tree was deduced from the alignment of the B subunit Gyrase sequences obtained using the phylogeny software PhyML 3.0 (www.phylogeny.fr^58^). The accession number of the sequences included in the phylogenic tree are, from top to down of the tree (in brackets, the percentage of identity with the sequence of the isolated bacterium): FJ238497.1 (91%); AY101823.1 (95%); AY101787.1 (97%); AB473087.1 (98%); AY101791.1 (99%); EU616627.1 (92%); AY101789.1 (94%); FJ238494.1 (91%); AY101822.1 (91%); AY101820.1 (90%); AY101772.1 (86%).

BLASTN analysis of gyrB genes obtained from strains isolated in the digested blood contained in the crop of *H. verbana* (Fig. 1a) assigned them to the genus *Aeromonas*. Multiple alignments of the gyrB genes (Fig. S1) and the resulting phylogenetic tree confirmed that this bacterial strain is *Aeromonas veronii biovar sobria* (Fig. 1b).

### Production of antibacterial substances by *Aeromonas sp*

**Figure 2:**
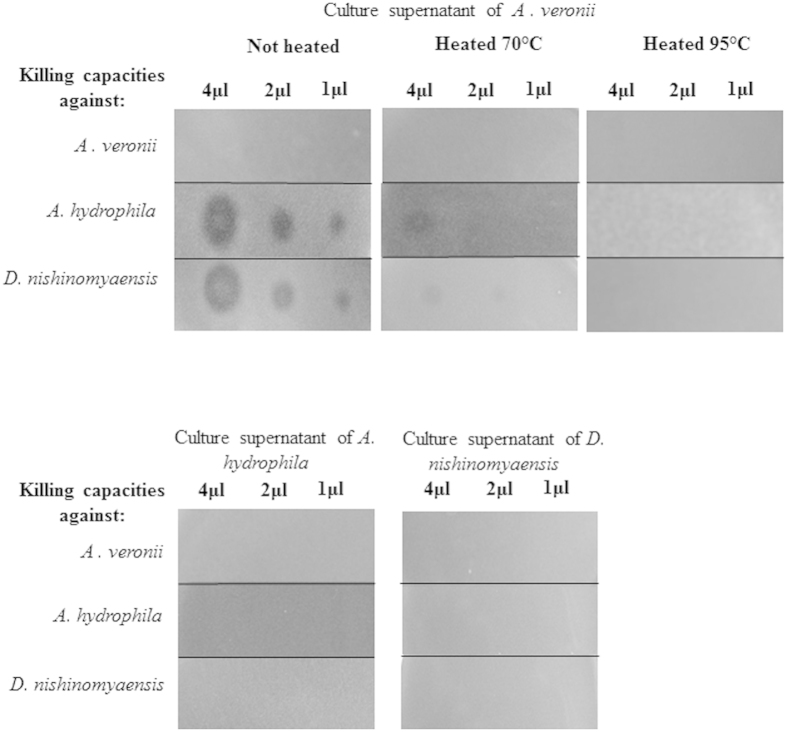
Antimicrobial properties of the substances produced by two phylogenetically related *Aeromonas* species. After a peptidic prepurification, the culture supernatants of *A. veronii, A. hydrophila* and *D. nishinomyaensis* were tested for their antimicrobial properties against the two *Aeromonas* species and against the Gram positive *D. nishinomyaensis.* Activities that appear as zones of growth inhibition, are lost when the extracts are heat-denatured with a complete denaturation at 95 °C.

The *Aeromonas veronii* free culture supernatant, which reflects what might be released by the bacteria into the lumen of the host gut, was tested for its activities against two other bacteria living in the environment of the leech. A dose-dependent inhibition of the bacterial growth was observed against the Gram-positive *D. nishinomiyaensis* and the Gram-negative *A. hydrophila* but not against *A. veronii* itself (Fig. 2). Activities were lost when the pre-purified extracts were heat denatured suggesting a proteic nature of the antibiotic substances produced by *A. veronii*. In order to check whether *A. hydrophila* and *D. nishinomyaensis* reciprocally produce anti-*A. veronii* substances, the same extraction procedure was applied to their culture supernatants. No activities against the other *Aeromonas* strain or against *D. nishinomyaensis* were detected in the supernatant culture of *A. hydrophila* or *D. nishinomyaensis* (Fig. 2).

### Nature of the antibiotics produced by the symbiont

**Figure 3:**
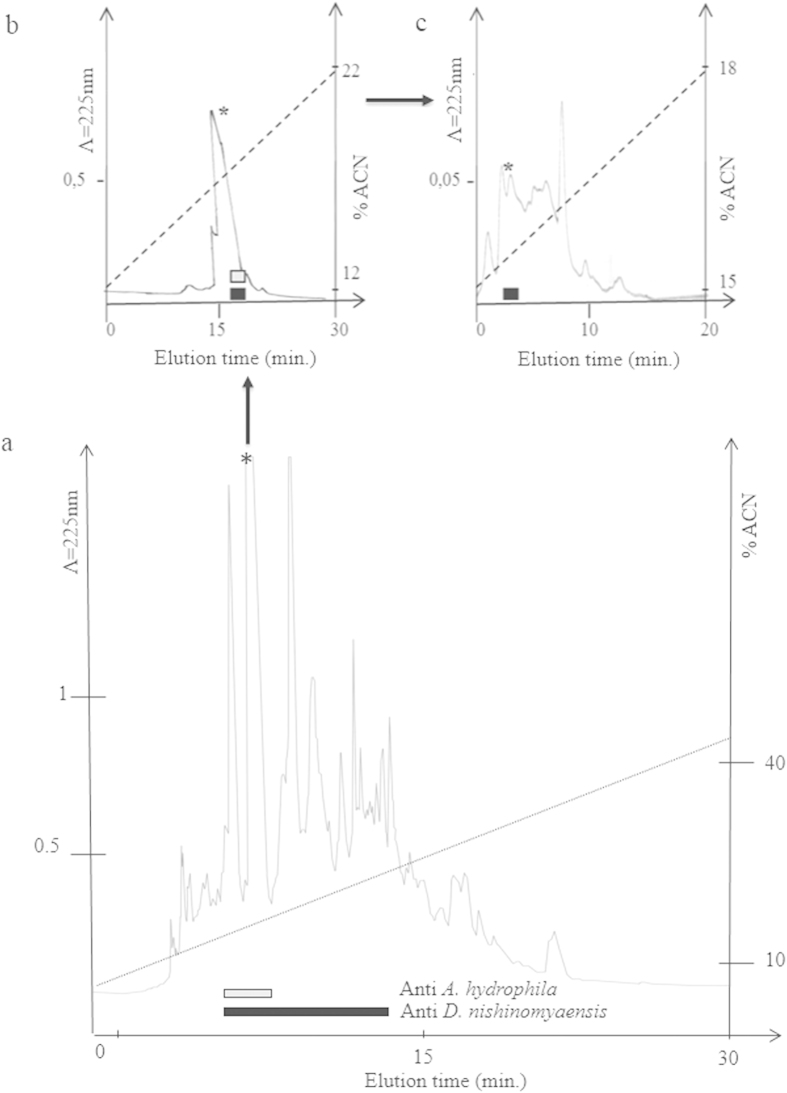
Purification of the antimicrobial peptides secreted by *Aeromonas veronii.* The eluted at 100% acetonitrile (ACN) upon solid phase extraction was loaded onto a C18 column (250 × 4 mm, Vydac). Elution was performed with a linear gradient of acetonitrile in acidified water (dotted line), and absorbance was monitored at 225 nm. Each individually collected fraction was tested for its antimicrobial activity against *A. hydrophila* (white bar) and *D. nishinomyaensis* (black bar). (**a**) Fractions containing antimicrobial active substances were further purified by two additional RP-HPLC (**b,c**). The fraction that is still active at the third step of chromatography was further analyzed by mass spectrometry (Fig. S2).

**Figure 4:**
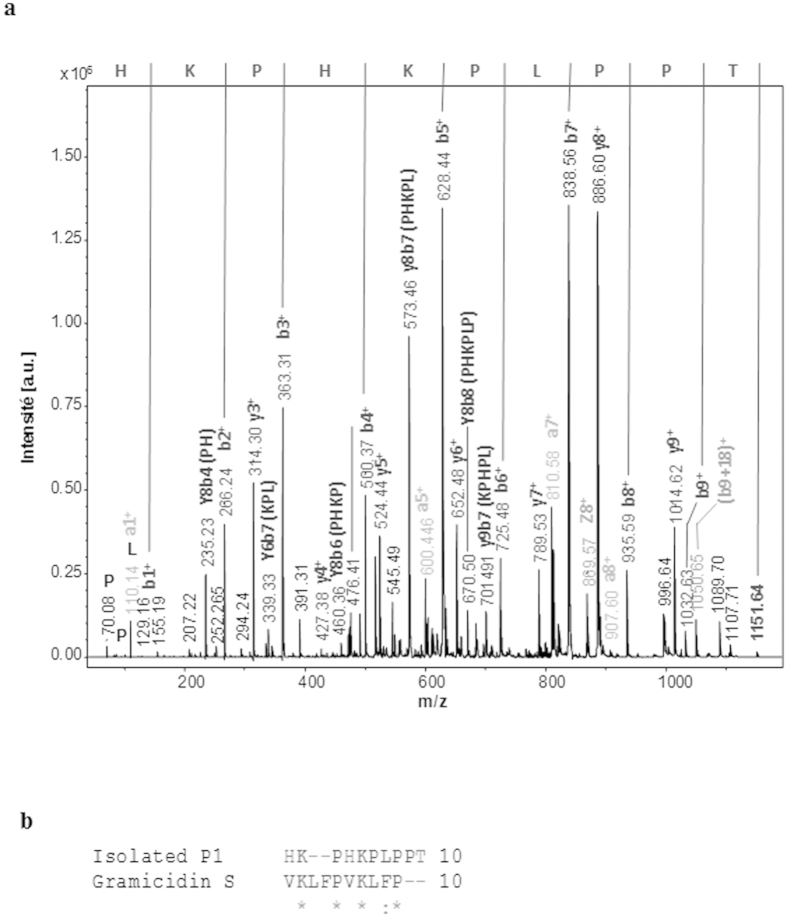
Identification of the antimicrobial peptide produced by *Aeromonas veronii*. (**a**) MS/MS MALDI-TOF spectrum of the 1151.74 m/z fragmented ion. The spectrum was annotated and *de novo* sequencing gives as sequence: HKPHKPLPPT. (**b**) Alignment of the antibiotic peptide produced by *Aeromonas veronii* with gramicidin S, the antibiotic peptide from *Bacillus brevis*.

Series of chromatography from the pre-purified extract were then undertaken in order to purify the antibacterial substance secreted by *A. veronii* (Fig. 3). The third RP-HPLC fractions that were still active (Fig. 3c) were analyzed by mass spectrometry (MALDI TOF/TOF) (Fig. S2). Two major peaks with m/z of respectively 1151. 64 (P1) and 1298.67 (P2) were detected (Fig. S2). The MS/MS experiments performed on all of them clearly showed that these peaks are related to peptides due to the presence of characteristic ions like the ammoniums. The MS/SM spectra in positive mode of ions at m/z 1151.64 (peptide P1) and 1298.67 (peptide P2) are presented in Fig. S2. *De novo* sequencing was then performed by MS/MS (Fig. 4a). Analyses using the Biotools and Rapid *de novo* sequencing 3.1 software was performed with a tolerance of 0.2 Da in MS and MS/MS as well. Only P1 (m/z of 1151.64) gave a clear sequence: HKPHKPLPPT (Fig. 4b). Analyses using the Antimicrobial Peptide Calculator and Predictor (The Antimicrobial Peptide Database: http://aps.unmc.edu/AP/main.php) of P1 (Fig. 4b) revealed some identities (38%) with gramicidin S, an also 10-amino acids antibiotic produced by the bacterium *Bacillus brevis*.

### Antibiotics produced by the host gut

**Figure 5:**
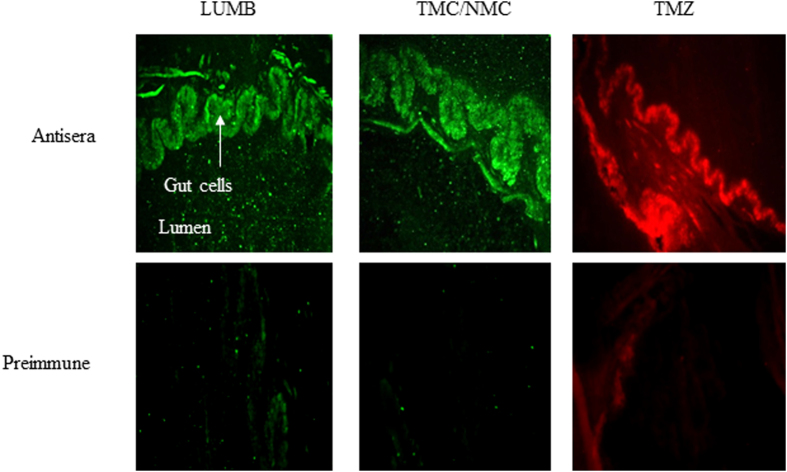
Immunohistochemistry data illustrating the presence of the three AMPs into the intestinal cells of starved leeches. Pre-immune sera are used as negative controls.

**Figure 6:**
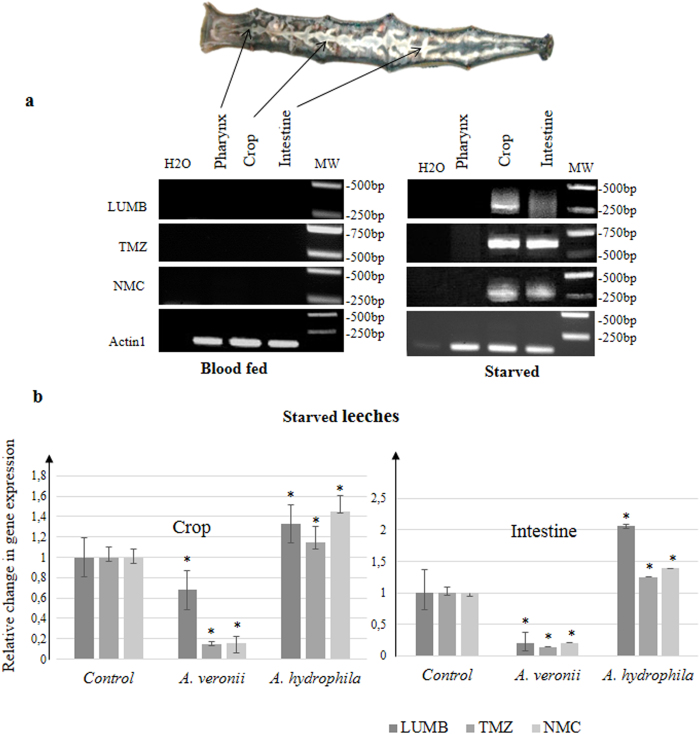
Identification and differential expression of the genes encoding AMPs along the digestive tract of starved *versus* fed leeches. **(a**) RT-PCR by using forward primer (TMC/NMC, TMZ or LUMB) with an oligodT was performed on RNA extracted from different dissected parts of the digestive tract one week after feeding and after one month of starvation. Amplified products were sub-cloned and sequenced. (**b**) NMC, LUMB and TMZ gene expressions were assessed by qRT-PCR in the intestine and the crop of starved leeches incubated with alive bacteria (*A.* *veronii* or *A. hydrophila)* or without (control) for 3 days. qRT-PCR results are presented as graphics. Graphics represent the best results of three independent experiments that displayed similar variations; p-values from Student’s T-tests were calculated between the different conditions, based on experimental measurements performed in triplicate (*p < 0.05).

Immunohistochemistry experiments showed a labeling of the gut epithelium for each anti-AMP antibody tested (Fig. 5). Because the anti-TMC antibody cross-reacts with NMC (Fig. S3), PCR using a forward primer common to NMC and TMC (Fig. S4), and an oligodT as reverse primer, were performed on RNA extracted from the leech intestine to identify the correct target. Amplifications were performed in parallel with the TMZ and LUMB primers (Fig. 6a). The sequencing of the amplified products allowed identifying that the digestive tract of *H. verbana* synthesizes at least three AMPs: TMZ, NMC (and not TMC) and LUMB.

### Influence of the symbiont load on the expression of host antibiotics

The possible correlation between host immunity and the symbiont load was also investigated from the samples that allowed identifying the nature of the AMPs produced by the leech gut. The gene expression of the three leech AMPs was quantified by semi-quantitative RT-PCR along the digestive tract according to the known spatio-temporal dynamics of the symbiont inside the gut (Fig. 6a)^6^. Indeed, we knew from the previous works of Graf and colleagues that the abundance of *Aeromonas* in the gut is low (~5 × 10^5^ CFU/ml of crop contents) before consumption of the blood meal (starved leeches), immediately increases by roughly three orders of magnitude after feeding and then declines by one week to return to an almost undetectable level^6^. Data indicated that the three AMP genes are expressed in both the intestine and the crop of starved leeches but not in the pharynx, the only site where the symbionts do not accumulate (Fig. 6a). By contrast, no transcripts were detected in the gut of freshly fed leeches suggesting an inhibition of the transcription process by the abundantly present symbiont.

The influence of the microbial flora present in the environment of the leech on gut immunity was also explored. Live *A. hydrophila* or *A. veronii* were added to the water of starved leeches (*i.e.* with a low load of symbionts) and the synthesis of AMPs by the host gut was quantified by quantitative RT-PCR (Fig. 6b). Under these conditions, the gene expression of the three leech AMPs was enhanced in the digestive tract of starved leeches incubated with *A. hydrophila* whereas it was repressed by the presence of the symbiont *A. veronii* in the water. *A. veronii* and *A. hydrophila* differentially influenced the antibacterial immunity of the gut in the medicinal leech *H. verbana*.

## Discussion

**Figure 7:**
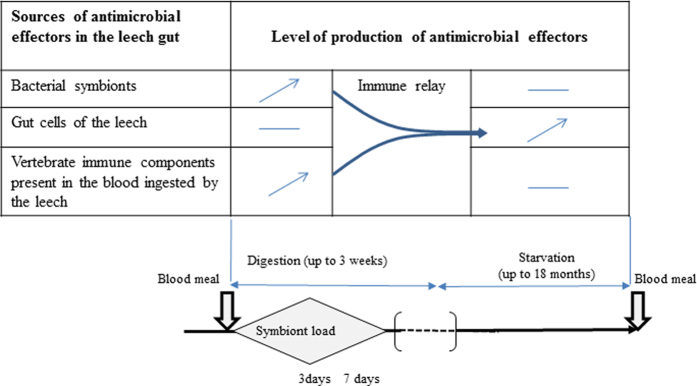
Hypothetical scheme resuming the role of antibiotics in the immune interaction between the leech and its gut symbiont. After blood feeding, *Aeromonas* that permanently persists at low level in the gut gains access to nutrient and starts proliferating. This bacterium becomes rapidly the numerically dominant bacteria of the crop by using various strategies to eliminate the other microorganisms such as: (**i**) the production of AMPs that act as anti-competitor agents, (**ii**) the protection from host phagocytic cells^21^ and (**iii**) a resistance to the vertebrate complement still active in the ingested blood. These different bacterial factors also provide antimicrobial immunity to the crop epithelium which stops producing its own immune factors *i.e.* NMC, TMZ, and LUMB. As the blood is being digested, the symbiont abundance in the crop as well as the so called “procured immunity” gradually declines^60^. AMPs encoded by the leech genome may then take the immune relay and warrant the antimicrobial defense of both the gut and the *Aeromonas* symbiont now less abundant and thus less able to compete with other bacteria through interference competition. Based on the natural feeding/starvation cycle of the leech, this scheme presumably repeats itself with each blood meal during the long lifespan of the medicinal leech.

Altogether our study highlights the roles of antibiotics in the interaction between *H. verbana* and its *Aeromonas* symbiont (Fig. 7). We evidenced that both the leech and its gut symbiont produce antibiotic substances that presumably (i) provide them with reciprocal protection against invasive bacteria and (ii) contribute to the unusual simplicity of the gut microflora of the leech. This immune benefit substantially reinforces the evidence of an evolutionarily stable association between *H. verbana* and *A. veronii*.

Siddall and collaborators showed that the gut of different species of medicinal leech houses different *Aeromonas s*pecies^37^. Our model *H. verbana* is most often associated with *A. veronii* whereas the two other medicinal leeches, *H. medicinalis* and *H. orientalis,* were reported to carry *A. hydrophila* and *A. veronii* and/or *A. jandaei* respectively. More recently, the same authors suggested a more complex system than initially determined: they observed an intriguing lack of evolution between the phylogenetic distribution of *Aeromonas sp.* and the phylogeny of the leeches caught in the wild^38^. Because of these observations, the identification of the *Aeromonas sp.* present in the gut of our model appeared as an essential prerequisite to our study. Our data confirmed the association of *H. verbana* with *A. veronii* a versatile bacterium that seems to have a propensity for colonizing the gut of hematophagous animals^39^.

Because multiple enteric bacteria are known to secrete and use AMPs as anti-competitor agents against other bacteria^31^, such a scenario was explored for the *A. veronii* strain isolated from the leech gut. *M. hirudinis,* the second major mutualistic bacterium of the leech gut, was not included in this scenario because of the difficulty to cultivate it and to use it for standardized antimicrobial assays (anaerobe). Such studies would have to be performed to draw a more complete picture of the role played by antibiotics in the interactions between the symbionts and with their host in the digestive tract of the leech.

Interestingly, our data show that *A. veronii* produces substances active against the phylogenetically related species, *A. hydrophila,* and the unrelated Gram-positive *D. nishinomyaensis*, another bacterium found in the freshwater habitat of *H. verbana.* By contrast, under the same conditions, the culture supernatant of *A. hydrophila* does not display any bacteria-killing capacities neither against *D. nishinomyaensis* nor against *A. veronii*. This latter observation is in line with the previous work of Messi and collaborators who observed that *A. hydrophila* antibacterial activity emerged with phylogenetically unrelated genera or species such as *Staphylococcus* and *Listeria* strains^40^. Here, we describe the nature of the antibiotics produced by *A. veronii,* which present homologies with gramicidin S, a cyclic antibiotic identified from the bacterium *Bacillus brevis*^41^. Searching for the obtained sequences in the genome of *Aeromonas* (J. Graf personal communication) was unsuccessful. This observation may suggest that these molecules, like gramicidin S, rather than being gene-encoded AMPs, might be produced through a non-ribosomal pathway that involves multi-enzyme complexes called peptide-synthetases^42^. This is the first description of an antibiotic produced by an *Aeromonas* strain. Further biochemical investigations on the secondary/tertiary structures and the mode of action of this peptide would be necessary to explore the potential use of such molecules as therapeutic agents against *Aeromonas hydrophila* a causative bacterium of infectious diseases in humans and fishes^16^,^17^. Moreover, in order to extrapolate our data to other *Aeromonadaceae*, it would be interesting to screen multiple strains isolated from different medicinal leeches caught in the wild (and not farm-raised under controlled conditions), to test them against wider range of bacteria and to determine whether they produce the antibacterial peptide described here.

From the data presented here, the preponderant association *H. verbana*/*A. veronii* found in nature may be explained by the powerful chemical weapon produced by *A. veronii* that helps clear the milieu from its direct competitor, *A. hydrophila.* This chemical warfare may also explain why the association of these two *Aeromonadaceae* has never been reported in the crop of any medicinal leeches, whereas both bacteria are available in the habitat and are able to colonize the digestive tract^11^.

The host-symbiont association confers an exclusive trophic niche to the bacterial partner as well as an efficient defense system against other microorganisms in the leech gut. The access to such advantages should select for strong competitive behavior between the two *Aeromonas* species, both resistant to the antibiotic substances produced by the leech. However, such competitiveness might be manifested in two ways: (i) through indirect resource competition or (ii) through direct interference competition. Here, we evidenced that the *A. veronii* strain isolated from *H. verbana* may have been selected to produce antibiotics that serve as anti-competitors, thereby ensuring their successful establishment into the leech crop. This strategy evokes the use by Enterobacteria of antimicrobial bacteriocins as weapons for microbial competitions/survival in the mammalian digestive tract^43^. Because *A. veronii* symbionts are vertically but also horizontally transmitted in *H. verbana*, it can be hypothesized that this dual mode of transmission allows for the selection of symbionts efficient at interference competition with other potentially pathogenic microbes while keeping their virulence towards their host in “evolutionary check”^44^,^45^. Essentially, such a hypothesis is based on the assumption of a trade-off between resource competitiveness (*i.e.* the ability of a given strain to deplete resource stocks to lower levels) and interference competitiveness^46^. Thus, a strongly interfering strain such as *A. veronii* might outcompete other bacterial strains at blood meals (when resource levels within the gut are high and not limiting) while antibiotic substances produced by the leech in-between blood meals might help prevent colonization by some pathogenic, resource-competitive bacteria. Horizontal transmission of microbiota can reinforce this mechanism in two ways: (i) more interference-competitive symbionts might be “recruited” over time with higher transmission among leeches and (ii) already resource-competitive strains might be prevented from becoming better at interference competition through selection for more virulence induced by numerous founder strains and the milker-killer dilemma^47^.

Several studies evidenced that host AMPs are involved in controlling and shaping the symbiotic microflora^24^,^48^,^49^. Our present data show that the digestive tract of *H. verbana* produces at least three AMPs: TMZ, NMC and LUMB that can be secreted into the extracellular medium as previously demonstrated^35^. The production of other AMPs cannot be excluded and purification from gut extracts should be performed to assess this possibility. Once synthesized by the intestinal cells, the three antibiotics are probably released into the lumen where they exert their antimicrobial properties. Our previous studies showed that none of these leech molecules are active against *Aeromonas.* However, they kill Gram-positive bacteria such as *Staphylococcus sp,* a resident skin commensal of vertebrates and *D. nishinomyaensis* a bacterium primary isolated from water and vertebrate skin. This observation probably reflects an adaptation of the leech to the microbes it is exposed to. Indeed, when *H. verbana* bites its prey for feeding, it is mostly exposed to Gram positive bacteria which represent the major microbial community covering the vertebrate skin^50^. NMC and TMC belong to the macin family^34^. Although they present common structural features, these two AMPs behave slightly differently^51^. Compared to TMC, the activity of NMC against some Gram positive bacteria is stronger and less salt-sensitive. Over the course of a single meal, the leech can consume over five times its own body weight in blood leading to a strong increase of the osmolyte concentrations into the digestive tract^11^. Thus, NMC was probably selected instead of TMC to cope with the variation in salt concentrations in the gut environment. By eliminating microorganisms other than *Aeromonas*, host AMPs protect and help the symbiont in colonizing the crop; in return, the latter may provide defense against pathogens potentially brought by the ingested blood, as does the gut microflora that protects the mammalian gut, through AMP-mediated interference competition among microorganisms^23^.

In mammals, several studies have evidenced that the gut microbiota shapes the intestinal immune response^26^,^52^. Our data showed an influence of the symbiont load during blood feeding, on the gene expression of NMC, TMZ and LUMB that are repressed when *A. veronii* are highly abundant in the crop while induced when the symbiont load is low. From a proximal point of view, these observations are reminiscent of the suppression of the expression of AMP genes by the resident gut microflora in the digestive tract of *Drosophila melanogaster*. In the fruit fly, the homeobox gene Caudal (Cad) interferes with the activation of AMP genes mediated by a NF-kB related transcription factor called relish. Gene silencing of Cad leads to an overproduction of AMPs and a strongly altered composition of the microflora^53^. Analyses of the *H. verbana* EST database reveal the presence of Cad and NF-kB orthologs (unpublished data) suggesting that such mechanism might be involved in the inhibition of AMP gene expression observed in our system. It will be interesting in the future to determine whether this inhibition is correlated to the biofilm formed by the gut symbionts in the week following the blood meal^6^. From an ecological point of view, the significant decrease in host immune gene activation after feeding may also suggest an energetic trade-off between digestion and immunity in the leech that ingests in few minutes five times its bodyweight in food. The impressive rising of the *Aeromonas* population after feeding coupled with the bacterial killing capacity of the symbiont may allow the host which has shut down its immune defense, to delegate its gut immunity to its symbiont and to reallocate energy to the digestion.

Moreover, it cannot be excluded that the differential gene expression of the leech AMPs observed here in the gut of animals exposed to either *A. veronii* or *A. hydrophila* added to their water environment, might result not only from bacteria that enter the gut, but also from a peripheral signal induced by the bacteria in contact with the leech skin. Indeed, our recent observations (unpublished data) suggest that leeches bathing in freshwater enriched in bacteria, present an immune stimulation not only in the digestive tract but also in multiple organs including some not directly exposed to the environment such as the central nervous system. Further investigations in this direction are planned to clearly establish the impact of *A. veronii* on the peripheral and neural immunity of the host. An effect of the gut symbiont that is not restricted to the digestive tract might be assumed taking into account the growing evidence of the multiple roles that exert symbiotic bacteria on their host physiology and adaptation^54^,^55^.

## Methods

### Leeches and bacteria

Farm-raised *Hirudo verbana* (Ricarimpex, France) wrongly commercialized as *Hirudo medicinalis*^37^ were maintained in the lab in autoclaved artificial pond water (38 mg/liter of Instant Ocean Salts) at 20 °C. Experiments were performed 3 days after blood feeding or after 1 month of starvation.

To study the impact of the microbial environment, live bacteria (*Aeromonas veronii, Aeromonas hydrophila* and *Dermacoccus nishinomiyaensis* (formerly known as *Micrococcus nishinomyaensis*), at 1.6 × 10^8^ CFU/ml), were added to the tank water of starved leeches. 72 h later, leeches were anaesthetized for 20 min at 4 °C in 10% ethanol and dissected. The Gram-positive *D. nishinomiyaensis* and the Gram-negative *Aeromonas hydrophila* bacteria strains were previously isolated and identified from the water environment of *H. verbana*^34^.

### Isolation and identification of the gut symbiont

Symbionts were isolated from the intraluminal fluid of the crop obtained aseptically from a leech anaesthetized for 20 min at 4 °C in 10% ethanol. The collected intraluminal fluid was spread on a culture plate of Luria Bertoni (LB) – agar – ampicillin (100 μg/ml, Sigma Aldrich). Ampicillin served as an *Aeromonas* selective agent (http://www.bacterio.net/aeromonas.html). Gram staining was performed on the isolated colonies^56^. Bacteria were further characterized by sequencing the gyrB gene (which encodes the B-subunit of DNA gyrase, a type-II DNA topoisomerase), a suitable marker for phylogenetic studies of the genus *Aeromonas*^57^. After isolation, one colony was grown in liquid LB overnight at 37 °C, under agitation. The bacterial pellet obtained after centrifugation was re-suspended in 300 μl of sterile Ringer (in mM: NaCl 115; KCl 4; CaCl_2_ 1.8; glucose 10 and Tris maleate 10, buffered to pH 7.4 with NaOH) and boiled for 10 min. The extract obtained was diluted in 3 ml of Tris-HCl 5 mM, pH 8.5; 2 μl was used as matrix for PCR amplification, using the primers gyrB3F (5′-TCCGGCGGTCTGCACGGCGT-3′) and gyrB14R (5′-TTGTCCGGGTTGTACTCGTC-3′), with 1 unit of Taq polymerase (GoTaq, Promega) in 1.5 mM of MgCl2. The cycling parameters were 95 °C for 2 min, then 40 cycles at 95 °C for 30 s 55 °C for 30 s and 72 °C for 1 min 20 s, and a final step of 10 min at 72 °C. The PCR product, loaded on an agarose gel, revealed a single band of the expected size, which was purified with the Nucleospin Extract II kit (Macherey-Nagel), cloned in pGEM T-easy vector (Promega) according to the protocol provided by the manufacturer and transformed into competent *Escherichia coli* JM 109 cells (Promega). DNA plasmids were sequenced with FM13/RM13 universal primers from Genoscreen. The sequence obtained was aligned using ClustalW with the most similar sequence from *Aeromonas veronii biovar sobria* (Genbank accession number: AY101791). The sequences that best match with ours in Blastn analyses were retrieved from GenBank and used for the construction of a phylogenetic tree using PhyML 3.0 (www.phylogeny.fr^58^).

### Isolation and identification of the antibiotic produced by the symbiont *Aeromonas veronii*

#### Purification of the antimicrobial peptide

One colony of *A. veronii* was cultured in LB medium for 12 h at 37 °C under agitation, and then centrifuged at 4500 g for 40 min, 4 °C. Peptides were pre-purified by loading 800 ml of supernatants onto Sep-Pak C18 Vac cartridges (Waters). Elution steps were performed with acetonitril (ACN) in 0.01% trifluoroacetic acid (TFA) water. The pre-purified fractions were then lyophilized, reconstituted in 10 ml of water and tested for their antimicrobial properties. Tests were also performed after heating the samples at 70 °C or 95 °C for 15 min. The same pre-purification procedure and antimicrobial assays were applied to the culture supernatant of isolated *A. hydrophila* and *D. nishinomyaensis*.

All the purification steps were carried out by reversed-phase high pressure liquid chromatography (RP-HPLC) on a Perkin-Helmer™ SERIES 200 HPLC system. In the first step, aliquots were subjected to RP-HPLC on a Sephasyl C18 column (250 × 4.6 mm, 218TP54 Vydac™). Elution was performed with a linear gradient of 1% ACN/0.05% TFA /min at a flow rate of 1 ml/min. Fractions were collected percentage per percentage in polypropylene tubes, lyophilized, reconstituted in pure water and tested for antibacterial activity. Active fractions were further loaded onto a C18 column (250 × 4.6 mm, 218TP54 Vydac™) with a linear gradient of 0.33% ACN/0.05% TFA/min at a flow rate of 1 ml/min. Fractions were collected and treated as above. For the last step of purification, active fractions were loaded onto a C18 column (250 × 2.1 mm, 218TP52, Vydac™) with a linear gradient of 0.16% ACN/min at a flow rate of 0.2 ml/min. Fractions corresponding to absorbance peaks were collected and tested for antibacterial activity.

#### Identification of the antimicrobial peptide

1 μl of each sample was spotted on a MTP 384 target plate polished steel (Bruker Daltonics), together with 1 μl of HCCA matrix (cyano-4-hydroxycinnamic acid, 10 mg/ml Sigma Aldrich). Mass spectrometry analyses were performed in positive reflector mode with an UltraFlex II MALDI-TOF/TOF instrument (Bruker Daltonics, Bremen, Germany) equipped with a Smartbeam laser having a repetition rate up to 200 Hz and controlled by FlexControl 3.0 (Build184) software (Bruker Daltonics, Bremen, Germany). The spectra were treated with FlexAnalysis 3.0 (Build 96) software.

A total of 2000 spectra were acquired for each sample. Interesting peptides were fragmented in MS/MS (positive ion mode) and spectra were annotated with Biotools 3.1 (Bruker Daltonics) and Rapid de novo sequencing 3.1 (Bruker Daltonics, Bremen).

#### Antibacterial assays

After each purification step, antibacterial activity was monitored against *A. veronii, A. hydrophila* and *D. nishinomyaensis* by a solid plate assay and by a liquid assay detailed in the electronic supporting information.

#### Gene expression analyses and identification of the AMP synthetized by the gut of *Hirudo verbana*

##### RNA extraction

Three medicinal leeches starved for 1 month and 3 leeches fed 3 days earlier were anaesthetized for 20 min in ethanol 10% and then dissected. The three parts that form the digestive tract (pharynx, crop, intestine) were separately collected in 1 ml of Qiazol lysis reagent (Qiagen). Tissues were grinded in Qiazol with a polytron, and RNAs were extracted according to the manufacturer’s instructions. Total RNAs were treated with RQ1Dnase1 (Promega, France) to prevent contaminations with genomic DNA.

For each sample, first strand cDNA was generated from 2 μg of total RNA and superscript II reverse transcriptase (RT) kit (Invitrogen, France) in a final volume of 20 μl.

##### Semi-quantitative RT-PCR and cDNA cloning of the AMPs produced by the digestive tract of Hirudo verbana

GenBank accession number of the studied genes: LUMB: ABW97520.1; TMC EU149766.1; NMC: EU156754.1; 18S: U67141.1 and actin1: DQ333330.1.

Amplifications of the genes of interest were realized with 2 μl of cDNA generated with oligodT using a sense primer (LUMB: 5′-AGATGGAGGAGGAAATTGAAGAACT-3′, Tm = 50 °C; TMZ: 5′-GACCATCACCACGACCATGGGCACG-3′, Tm = 58 °C; TMC/NMC: TGTTTCGAAGATTGGAGTCGTTGTTCG-3′, Tm = 53 °C) and an oligodT_34_ (CGAGTCGACATCGATCG(T)18) as antisense primer. Actin1 was amplified as a reference gene (sense primer: 5′-GAACACCCAGTCCTCCTGAC-3′; antisense primer: 5′-GCTGTCCTGTCCCTTTATGC-3′; Tm = 59 °C). The conditions of DNA amplification included an initial denaturation step of 2 min at 95 °C, 40 cycles of 30 s at 95 °C, 30 s at Tm, and 30 s at 72 °C, and finally 3 min at 72 °C. The PCR products were loaded onto a 1.5% agarose gel. All PCR products were ligated into the PGEMT easy vector (according to the protocol provided by the manufacturer) and transformed into competent *Escherichia coli* JM 109 cells (Promega). Plasmids DNA were sequenced with a FM13/RM13 sequencing kit (Pharmacia Biotech) according to the manufacturer’s instructions.

##### Quantitative RT-PCR

Real time PCR were performed with the Quantitect SYBR Green PCR kit (Qiagen) by combining 1 μl of cDNA generated using random primers, 0.8 μM of each specific primer (NMC, LUMB, TMZ and 18S as reference), and 1μl of SYBR Green reagent in a final volume of 25 μl, using the previously described protocol^59^.

### Immunohistochemistry

The antibodies used to label slices of tissue were raised in the laboratory as already detailed in previous studies^34^. Because of the high conservation between NMC and TMC epitopes, the TMC antiserum cross-reacts with both targets (Fig. S3).

Leeches were fixed overnight in 4% paraformaldehyde. After dehydration, animals were embedded in paraplast and 7 μm sections were cut, mounted on poly-L-lysine-coated slides, and stored at 4 °C until use. Paraffin sections were re-hydrated and non-specific background staining was blocked as previously described^33^. Samples were then incubated with rabbit LUMB antibodies (1:200), rabbit TMC antibodies (1:800) or mouse TMZ antibodies (1:800) diluted in the AB solution (PBS containing 1% BSA 0.05% Triton 1% NGS 1% NDS 1% Ovalbumin) overnight at RT. Primary antibodies were removed and samples were incubated either with a FITC conjugated goat anti-rabbit secondary antibody or with a Texas Red conjugated goat anti-mouse secondary antibody. As a control, the immune-labeling procedure was carried out with the respective pre-immune sera. Slides were then mounted in glycergel (Dako) and examined using a fluorescent inverted microscope (Leica DMIRE2).

## Additional Information

**How to cite this article**: Tasiemski, A. *et al.* Reciprocal immune benefit based on complementary production of antibiotics by the leech *Hirudo verbana* and its gut symbiont *Aeromonas veronii*. *Sci. Rep.* **5**, 17498; doi: 10.1038/srep17498 (2015).

## Supplementary Material

Supplementary Information
