# Supplementary material for: Reciprocal immune benefit based on complementary production of antibiotics by the leech Hirudo verbana and its gut symbiont Aeromonas veronii
Source: Sci Rep. 2015 Dec 4;5:17498. doi: 10.1038/srep17498 (PMC4669451; doi:10.1038/srep17498)
Supplement: Supplementary Information [file srep17498-s1.pdf]

Reciprocal immune benefit based on complementary production of antibiotics by the leech *Hirudo verbana* and its gut symbiont *Aeromonas veronii*

Aurélie Tasiemski [a*], François Massol [a], Virginie Cuvillier-Hot [a], Céline Boidin-Wichlacz [a], Emmanuel Roger [c], Franck Rodet [b], Isabelle Fournier [b], Frédéric Thomas [d], Michel Salzet [b*]

Supplementary Figures

Figure S1: ClustalW alignment of the gyrB PCR product amplified from the isolated strain with the GyrB gene of *Aeromonas veronii biovar sobria*.

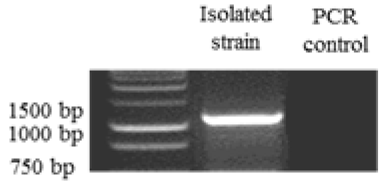


```
A.schubert       ----------------------------GCCGTCAGGGCCATGTCTACGAGCAGACCTAC
A.eucrenop       ----------------------------GTCGTAACGGGCACGTCTACGAGCAGACCTAT
A.salmonic       -TCTCTGACAAGCTGTTGCTGACCATTCGTCGTAACGGCCATGTCTACGAGCAGACCTAT
A.bestiaru       ----------------------------GTCGCAACGGCCATGTCTACGAGCAGACCTAT
A.media          CTCTCTGACAAGCTGCTGCTGACCATACGTCGCAACGGCCATGTCTACGAGCAGACCTAT
A.jandaei        ----------------------------GTCGTAACGGCCACGTCTACGAACAGACCTAT
A.hydrophi       ----------------------------GTCGCAACGGCCATGTCTACGAGCAGACCTAT
A.allosacc       ----------------------------GTCGTAACGGCCACGTCTACGAGCAGACCTAT
A.v.bv._ve       ----------------------------GTCGTAACGGTCACGTCTACGAGCAGACCTAT
A.sobria         ---------CAGCTGTTGCTGACCATTCGTCGTAACGGTCACGTCTACGAGCAGACCTAT
I.bacteria       ----------------------------GTCGTAACGGTCACGTCTACGAGCAGACCTAT
A.v.bv._so       ----------------------------GTCGTAACGGTCACGTCTACGAGCAGACCTAT
                                             * **  * **  ** ******** ********

A.schubert       AAGCTGGGCGAGCCGCAGGCGCCCCTCAAGCAGATCGGCGA---TGCCACCACCACGGGT
A.eucrenop       CACCTGGGCGAGCCGCAGGCACCGCTCAAGCAGATCGGCGACAGCTCTTCCGGTACCGGT
A.salmonic       CACCTGGGTGAGCCGCAGGCGCCGCTCAAGCAGATTGGCGACAGCAGCAGCGGCACGGGT
A.bestiaru       CACCTGGGTGAGCCACAGGCGCCGCTCAAGCAGATTGGCGACAGCAGCAGCGGCACGGGT
A.media          CACCTGGGTGAGCCGCAGGCACCGCTCAAGCAGATTGGCGA---CAGCACGGGCACAGGT
A.jandaei        CATCTGGGTGAGCCGCAGGCTCCGCTCAAGCAGATCGGTGA---CAGCACGGGTACCGGT
A.hydrophi       CACCTGGGTGAGCCGCAGGCACCGCTCAAGCAGATTGGCGA---CAGCACCGGCACCGGG
A.allosacc       CATCTGGGTGAGCCGCAGGCGCCGCTCAAGCAGATTGGCGA---CAGCACCGGCACCGGT
A.v.bv._ve       CATCTGGGTGAGCCACAGGCGCCGCTCAAGCAGATTGGCGA---CAGCACCGGCACCGGT
A.sobria         CATCTGGGTGAGCCGCAAGCGCCGCTCAAGCAGATTGGCGA---CAGCGCCGGCACCGGT
I.bacteria       CATCTGGGTGAGCCACAAGCGCCGCTCAGGCAGATTGGCGA---CAGCACCGGCACCGGT
A.v.bv._so       CATCTGGGTGAGCCACAAGCGCCGCTCAAGCAGATTGGCGA---CAGCACCGGCACCGGT
                  * ***** ***** ** ** ** **** ****** ** **             ** **

A.schubert       ACCGAGGTGCGCTTCTGGCCGAGCCCGACCATCTTCAGTGATACCCTGTTCCACTACGAG
A.eucrenop       ACCGAGGTGCGTTTCTGGCCGAGCCCGACCATCTTCAGCGATACCCTGTATCACTACGAG
A.salmonic       ACCGAAGTGCGCTTCTGGCCGAGCCCGACCATCTTTACCGATACCCTGTATCACTACGAG
A.bestiaru       ACCGAGGTGCGCTTCTGGCCGAGCCCGACCATCTTTACCGATACCCTGTATCACTACGAG
A.media          ACCGAAGTGCGCTTCTGGCCGAGCCCGACCATCTTTACCGATACCCTGTATCACTACGAG
A.jandaei        ACCGAAGTACGCTTCTGGCCAAGCCCGACCATCTTCAGCGATACCCTGTTCCACTACGAG
A.hydrophi       ACCGAAGTCCGCTTCTGGCCGAGCCCGACCATCTTCAGCGATACCCTGTTCCACTACGAG
A.allosacc       ACCGAAGTTCGCTTCTGGCCGAGCCCGACCATCTTTACCGATACCCTGTATCACTACGAG
A.v.bv._ve       ACCGAAGTTCGCTTCTGGCCGAGCCCGGCCATTTTCAGCGATACCCTGTTCCACTACGAG
A.sobria         ACCGAAGTTCGCTTCTGGCCGAGCCCTGCCATTTTCAGCGATACCCTGTTCCACTACGAG
I.bacteria       ACCGAAGTTCGCTTCTGGCCGAGCCCTGCCATTTTCAGCGATACCCTGTTCCACTACGAG
A.v.bv._so       ACCGAAGTTCGCTTCTGGCCGAGCCCTGCCATTTTCAGCGATACCCTGTTCCACTACGAG
                 ***** ** ** ******** *****  **** ** *  **********  *********

A.schubert       ATCCTGGCCAAGCGTCTGCGCGAGCTCTCCTTCCTCAACTCCGGCGTCTCCATCCGTGTC
A.eucrenop       ATCCTGGCCAAGCGTCTGCGCGAACTCTCCTTCCTCAACTCTGGCGTCTCCATCCGTCTG
A.salmonic       ATCCTGGCCAAGCGTCTGCGCGAGCTCTCCTTCCTCAACTCCGGTGTCTCCATCCGTCTG
A.bestiaru       ATCCTGGCCAAGCGTCTGCGCGAGCTCTCCTTCCTCAACTCCGGTGTCTCCATCCGTCTG
A.media          ATCCTGGCCAAGCGTCTGCGTGAGCTCTCTTTCCTGAACTCCGGTGTCTCCATCCGCCTG
A.jandaei        ATCCTGGCCAAGCGTCTGCGCGAACTCTCCTTCCTCAACTCCGGCGTTTCCATCCGTCTG
A.hydrophi       ATCCTGGCCAAGCGCTTGCGCGAGCTCTCCTTCCTCAACTCCGGTGTCTCCATCCGCCTG
A.allosacc       ATCCTGGCCAAGCGCCTGCGTGAGCTCTCCTTCCTCAACTCCGGTGTTTCCATTCGTCTG
A.v.bv._ve       ATCCTGGCCAAGCGCCTGCGCGAGCTCTCCTTCCTCAACTCCGGTGTCTCCATCCGTCTG
A.sobria         ATCCTGGCCAAGCGCCTGCGCGAGCTCTCCTTCCTCAACTCCGGTGTCTCCATCCGTCTG
I.bacteria       ATCCTGGCCAAGCGCCTGCGTGAGCTCTCCTTCCTCAACTCCGGTGTCTCCAGCCGTCTG
A.v.bv._so       ATCCTGGCCAAGCGCCTGCGTGAGCTCTCCTTCCTCAACTCCGGTGTCTCCATCCGTCTG
                 **************  **** ** ***** ***** ***** ** ** ****  **  *
```

```
A.schubert      TCGGACGAGCGTGATGGCCGTGAGGTGCACTTCTGCTACGAGGGCGGTATCAAGGCCTTC
A.eucrenop      CAAGACGAGCGCGATGGCCGCGAGGTGCATTTCTGCTACGAGGGTGGCATCGGGGCCTTC
A.salmonic      CAGGACGAGCGTGATGGCCGCGAGGTGCATTTCTGCTATGAGGGCGGTATCAAGGCGTTC
A.bestiaru      CAGGACGAGCGGGATGGCCGTGAGGTGCATTTCTGCTACGAGGGCGGTATCAAGGCGTTC
A.media         CTGGACGAGCGGGATGGCCGCGAGGCTCACTTCTGCTACGAGGGTGGCATCAAGGCCTTC
A.jandaei       ATGGATGAGCGTGACGGCCGTGAGGCGCACTTCTGCTACGAAGGCGGCATCAAGGCGTTC
A.hydrophi      CTGGATGAGCGTGACGGCCGCGAGGCGCACTTCTGCTACGAAGGCGGCATCAAGGCGTTC
A.allosacc      CTGGACGAGCGGGATGGTCGCGAGGCGCATTTCTGCTATGAGGGTGGTATCAAGGCGTTC
A.v.bv._ve      CAAGACGAGCGTGATGGCCGCGAGGCGCATTTCTGCTACGAGGGTGGTATCAAGGCATTC
A.sobria        CAAGACGAGCGTGATGGCCGCGAGGCGCATTTCTGCTACGAGGGTGGCATCAAGGCGTTC
I.bacteria      CAAGTCGAGCGTGATGGCCGCGAGGCGCATTTCTGCTACGACGGTGGCATCAAGGCATTC
A.v.bv._so      CAAGTCGAGCGTGATGGCCGCGAGGCGCATTTCTGCTACGAGGGTGGCATCAAGGCATTC
                   *  ***** ** ** ** ****  ** ******** ** ** ** ***  *** ***

A.schubert      GTCGAGTACCTGAACCAGAGCAAGACCCCGATCCACCAGAAGGTGTTGGACTTCAGCGCC
A.eucrenop      GTCGAATACCTGAACCAGAACAAGACTCCGATCCACCAGAAGGTGTTCCATTTCTCCACC
A.salmonic      GTTGAATACCTGAACCAGAACAAGACCCCGATCCACCCCAAGGTGTTCCACTTCTCCACA
A.bestiaru      GTTGAATACCTGAACCAGAACAAGACCCCGATCCATCCCAAGGTGTTCCACTTCTCCACA
A.media         GTCGAATACCTGAACCAGAACAAGACCCCGATCCATCCCAAGGTGTTTCACTTCACCACA
A.jandaei       GTTGAGTACCTGAACCAGAACAAAACCCCGATCCACCCGAAGGTGTTCCACTTCACCACC
A.hydrophi      GTTGAATACCTGAACCAGAACAAAACCCCGATCCACCCGAAGGTGTTCCACTTCACCACC
A.allosacc      GTTGAATACCTGAACCAGAACAAGACCCCGATCCACCCGAAGGTGTTCCATTTCACCACC
A.v.bv._ve      GTTGAGTACCTGAACCAGAACAAGACCCCGATCCACCCGAAGGTGTTCCACTTCACCACC
A.sobria        GTTGAATACCTGAACCAGAACAAGACCCCGATCCACCCGAAGGTGTTCCATTTCACCACC
I.bacteria      GTTGAATACCTGAGCAAGAACAAGACCCCGATCCACCCGAAGGTGTTCCATTTCACCACC
A.v.bv._so      GTTGAATACCTGAACCAGAACAAGACCCCGATCCACCCGAAGGTGTTCCATTTCACCACC
                ** ** ******* * *** *** ** ******** *  ********  * ***  * *

A.schubert      GAACAGGATGGCATCGGTGTCGAAGTCGCCATGCAGTGGAACGACGGCTATCAGGAAGGG
A.eucrenop      GAGCAGGACGGCATCGGCGTCGAAGTCCCCATGCAGTGGAACGATGCCTATCAGGAAGGG
A.salmonic      GAGCAGGACGGTATCGGTGTCGAAGTCGCCATGCAGTGGAACGATGCCTATCAGGAAGGG
A.bestiaru      GAGCAGGACGGCATCGGTGTCGAAGTCGCCATGCAGTGGAACGATGCCTATCAGGAAGGG
A.media         GAGCAGGATGGCATCGGCGTCGAAGTGGCGATGCAGTGGAACGACGCCTATCAGGAAGGG
A.jandaei       GAACAGGATGGCATCGGTGTGGAAGTCGCCATGCAGTGGAACGACGCCTATCAGGAAGGG
A.hydrophi      GAGCAGGATGGCATCGGTGTCGAAGTGGCGATGCAGTGGAACGACGCCTATCAGGAAGGG
A.allosacc      GAGCAGGATGGCATCGGCGTTGAAGTGGCGATGCAGTGGAACGACGCCTATCAGGAAGGG
A.v.bv._ve      GAGCAGGATGGTATTGGCGTTGAAGTGGCGATGCAGTGGAACGACGCCTATCAGGAAGGG
A.sobria        GAGCAGGATGGTATCGGCGTTGAAGTGGCGATGCAGTGGAACGACGCCTATCAGGAAGGG
I.bacteria      GAGCAGGATGGTATCGGCGTTGAAGTGGCGATGCAGTGGAACGACGCCTATCAGGAAGGG
A.v.bv._so      GAGCAGGATGGTATCGGCGTTGAAGTGGCGATGCAGTGGAACGACGCCTATCAGGAAGGG
                ** ***** ** ** ** ** *****  * ************** * *************

A.schubert      GTGTACTGCTTCACCAACAACATCCCGCAGCGTGACGGTGGTACCCACCTGGCCGGCTTC
A.eucrenop      GTGTACTGCTTCACCAACAACATCCCGCAGCGGGATGGCGGTAATCACCTGGTGGGCTTC
A.salmonic      GTTTACTGCTTCACCAACAACATCCCGCAGCGGGATGGCGGTACTCACCTGGTGGGTTTC
A.bestiaru      GTTTACTGCTTCACCAACAACATCCCGCAGCGGGATGGTGGTACTCACCTGGTGGGCTTC
A.media         GTCTACTGCTTCACCAACAACATCCCGCAGCGGGATGGGGGTACCCACCTGGTGGGCTTC
A.jandaei       GTCTACTGCTTCACCAACAACATTCCGCAGCGCGATGGTGGTACTCACCTCGTTGGTTTC
A.hydrophi      GTCTACTGCTTCACCAACAATATTCCGCAGCGCGATGGTGGTACCCACCTGGTGGGCTTC
A.allosacc      GTCTACTGCTTCACCAACAACATCCCGCAGCGGGATGGTGGTACTCACCTCGTTGGCTTC
A.v.bv._ve      GTCTACTGCTTCACCAACAACATCCCGCAGCGGGATGGTGGTACTCACCTCGTTGGCTTC
A.sobria        GTCTACTGCTTCACCAACAACATCCCGCAGCGGGATGGTGGTACTCACCTCGTTGGCTTC
I.bacteria      GTCTACTGCTTCACCAACAACATCCCGCAGCGGGATGGTGGTACTCACCTCGTTGGCTTC
A.v.bv._so      GTCTACTGCTTCACCAACAACATCCCGCAGCGGGATGGTGGTACTCACCTCGTTGGCTTC
                ** ***************** ** ******** ** ** ****  ***** *  ** ***

A.schubert      CGTGCCGCCCTGACCCGTACCCTGAACAGTTACATGGACAAGGAAGACTACAGCAAGAAG
A.eucrenop      CGTACCGCGCTGACCCGTACCCTCAACACCTATATGGACAAAGAGGACTACAGCAAGAAG
A.salmonic      CGTACCGCGCTGACCCGTACCCTCAATACCTATATGGACAAAGAGGATTACAGCAAGAAG
A.bestiaru      CGCACCGCGCTGACCCGTACCCTCAACACCTATATGGACAAAGAGGATTACAGCAAGAAG
A.media         CGTACCGCGCTGACCCGTACTCTGAACTCCTACATGGACAAAGAGGACTACAGCAAGAAG
A.jandaei       CGTACCGCGCTGACCCGTACCCTCAACTCCTACATGGAGAAAGAGGACTACAGCAAGAAG
A.hydrophi      CGTACCGCGCTGACCCGTACTCTGAACTCCTACATGGACAAAGAGGACTACAGCAAGAAG
A.allosacc      CGTACCGCGCTGACCCGTACTCTGAACTCCTACATGGACAAAGAGGACTACAGCAAGAAG
A.v.bv._ve      CGTACCGCGCTGACCCGTACTCTGAACTCCTATATGGACAAAGAGGACTACAGCAAGAAG
A.sobria        CGTACCGCGCTGACCCGTACTCTGAACTCCTATATGGACAAAGAGGACTACAGCAAGAAG
I.bacteria      CGTACCGCGCTGACCCGTACTCTGAACTCCTATATGGACAAAGAGGACTACAGCAAGAAT
```

```
A.v.bv._so      CGTACCGCGCTGACCCGTACTCTGAACTCCTATATGGACAAAGAGGACTACAGCAAGAAG
                **  **** *********** ** **    ** ***** ** ** ** ***********

A.schubert      GCCAAGACCTCCGCCAGCGGCGACGACGTGCGCGAAGGCCTGGTGGCGGTCATTTCGGTC
A.eucrenop      GCCAAATCGGCCGCCAGCGGCGACGACGTACGTGAAGGTCTGATTGCGGTCATCTCGGTC
A.salmonic      GCCAAGTCGGCCGCCAGCGGCGACGACGTGCGTGAAGGTCTGATTGCGGTCATCTCGGTC
A.bestiaru      GCCAAGTCGGCCGCCAGTGGCGACGACGTGCGTGAAGGTCTGATTGCGGTCATCTCGGTC
A.media         GCCAAGTCTGCCGCCAGTGGCGACGACGTCCGTGAAGGCCTGATTGCGGTCATCTCCGTC
A.jandaei       GCCAAATCTGCCGCCAGCGGTGATGACGTGCGTGAAGGTCTGATTGCCGTTATCTCCGTC
A.hydrophi      GCCAAGTCTGCCGCCAGTGGCGACGACGTGCGTGAAGGTCTGATTGCCGTTATCTCCGTC
A.allosacc      GCCAAGTCTGCCGCCAGTGGCGACGACGTACGTGAAGGTCTGATTGCCGTTATCTCCGTC
A.v.bv._ve      GCCAAGTCTGCCGCCAGTGGCGACGACGTGCGTGAAGGTCTGATTGCCGTTATCTCCGTC
A.sobria        GCCAAGTCTGCCGCCAGTGGCGACGACGTGCGTGAAGGTCTGATTGCCGTTATCTCCGTC
I.bacteria      GCCAAGTTTGCCGCCAGTGGCGACGACGTACGTGAAGGTGTGATTGCCGTTATCTCCGTC
A.v.bv._so      GCCAAGTCTGCCGCCAGTGGCGACGACGTACGTGAAGGTGTGATTGCCGTTATCTCCGTC
                *****     ******* ** ** ***** ** *****  ** * ** ** ** ** ***

A.schubert      AAGGTGCCGGATCCCAAGTTCTCCTCCCAGACCAAGGACAAGCTGGTCTCCTCGGAGGTG
A.eucrenop      AAGGTACCTGACCCTAAGTTCTCCTCCCAGACCAAGGACAAGCTGGTCTCTTCCGAAGTG
A.salmonic      AAGGTACCGGATCCCAAGTTCTCCTCCCAGACCAAGGACAAACTGGTCTCTTCCGAAGTG
A.bestiaru      AAGGTGCCGGATCCCAAGTTCTCTTCTCAGACCAAGGACAAGCTGGTCTCTTCCGAAGTG
A.media         AAGGTACCTGATCCCAAGTTCTCCTCCCAGACCAAGGACAAACTGGTCTCTTCCGAAGTG
A.jandaei       AAGGTGCCGGATCCCAAGTTCTCCTCCCAGACCAAGGACAAGCTGGTCTCTTCCGAAGTG
A.hydrophi      AAGGTGCCGGATCCCAAGTTCTCCTCCAGACCCAAGGACAAGCTGGTCTCTTCCGAAGTG
A.allosacc      AAGGTGCCGGATCCCAAGTTCTCCTCCCAGACCAAGGACAAGCTGGTCTCTTCCGAAGTG
A.v.bv._ve      AAGGTGCCGGATCCCAAGTTCTCCTCCCAGACCAAAGACAAGCTGGTCTCTTCCGAAGTG
A.sobria        AAGGTGCCGGATCCCAAGTTCTCCTCCCAGACCAAAGACAAGCTGGTCTCTTCCGAAGTG
I.bacteria      AAGGTGCCGGATCCCAAGTTCTCCTCCCAGACCAAAGACAAGCTGGTCTCTTCCGAAGTG
A.v.bv._so      AAGGTGCCGGATCCCAAGTTCTCCTCCCAGACCAAAGACAAGCTGGTCTCTTCCGAAGTG
                ***** ** ** ** ******** **     **** ***** ******** ** ** ***

A.schubert      AAGACCGCCGTCGAGCAGGCCATGGGCGACAAGCTGGCCGACTTCCTGCTGGAAAACCCG
A.eucrenop      AAGACCGCCCTTGAACAGGCGATGGGTGAGAAGCTGGCGGAGTTCCTGCTGGAAAACCCG
A.salmonic      AAGACCGCCGTTGAACAGGCGATGGGTGAGAAGCTCGGTGAGTTCCTGCTGGAAAACCCG
A.bestiaru      AAGACCGCCGTTGAACAGGCGATGGGCGAGAAGCTCGGCGAGTTCCTGCTGGAAAACCCG
A.media         AAGACCGCCGTTGAACAGGCAATGGGCGAGAAGTTGGCGGACTTCCTGTTGGAAAACCCG
A.jandaei       AAGACTGCTGTCGAGCAGGCGATGGGTGAGAAGCTGGCCGACTTCCTGCTGGAAAACCCG
A.hydrophi      AAGACCGCCGTAGAACAGGCGATGGGTGAGAAGCTGGCCGACTTCCTGCTGGAAAACCCG
A.allosacc      AAGACTGCCGTTGAACAGGCGATGGGTGAGAAGCTGGCCGACTTCCTGCTGGAAAACCCG
A.v.bv._ve      AAGACCGCCGTTGAGCAGGCGATGGGTGAGAAGCTGGCGGACTTCCTGCTGGAAAACCCG
A.sobria        AAGACCGCCGTTGAACAGGCGATGGGTGAGAAGCTGGCTGACTTCCTGCTGGAAAACCCG
I.bacteria      AAGACCGCCGTTGAACAGGCGATGGGTGAGAAGCTTGCCGACTTCCTGCTGGAAAACCCG
A.v.bv._so      AAGACCGCCGTTGAACAGGCGATGGGTGAGAAGCTTGCCGACTTCCTGCTGGAAAACCCG
                ***** **  * ** ***** ***** ** *** * *  ** ****** ***********

A.schubert      GGCGACGCCAAGATCGTGGTCAACAAGATAATCGACGCGGCCCGTGCCCGTGAGGCGGCC
A.eucrenop      GGCGATGCCAAGATCGTGGTCAACAAGATCATCGACGCGGCCCGTGCCCGTGAGGCGGCC
A.salmonic      GGCGATGCCAAGATAGTGGTCAACAAGATCATCGATGCGGCCCGTGCCCGTGAAGCGGCC
A.bestiaru      GGCGATGCCAAGATCGTGGTCAACAAGATCATCGATGCGGCCCGTGCCCGTGAAGCGGCC
A.media         GGCGACGCCAAGATCGTGGTCAACAAGATCATCGATGCGGCCCGTGCCCGTGAAGCGGCC
A.jandaei       GGCGATGCCAAGATCGTGGTCAACAAGATCATCGATGCGGCCCGTGCCCGTGAAGCAGCC
A.hydrophi      GGCGATGCCAAGATCGTGGTCAACAAGATCATCGATGCGGCCCGTGCCCGTGAGGCGGCC
A.allosacc      GGCGATGCCAAGATCGTGGTCAACAAGATCATCGATGCGGCCCGTGCACGTGAAGCGGCT
A.v.bv._ve      GGCGATGCCAAGATCGTGGTCAACAAGATCATCGATGCGGCCCGTGCCCGTGAAGCGGCC
A.sobria        GGCGATGCCAAGATCGTGGTCAACAAGATCATCGATGCGGCCCGTGCCCGTGAAGCGGCC
I.bacteria      GGCGATGCCAAGATAGTGGTCAACAAGATCATCGATGCGGCCCGTGCCCGTGAAGCGGCC
A.v.bv._so      GGCGATGCCAAGATCGTGGTCAACAAGATCATCGATGCGGCCCGTGCCCGTGAAGCGGCC
                ***** ******** ************** ***** *********** ***** ** **

A.schubert      CGCAAGGCGCGCGAGATGACTCGTCGCAAGGGTGCCCTGGACATCGCCGGCCTGCCCGGC
A.eucrenop      CGCAAGGCGCGTGAACTGACTCGCCGCAAGGGTGCACTGGACATCGCCGGTCTGCCCGGC
A.salmonic      CGCAAGGCCCGCGAGCTGACTCGCCGCAAGGGTGCGCTGGATATCGCCGGTCTGCCCGGC
A.bestiaru      CGCAAGGCGCGCGAGCTGACCCGCCGCAAGGGTGCGCTGGACATCGCCGGCCTGCCCGGC
A.media         CGCAAGGCCCGGGAACTGACTCGCCGCAAGGGTGCGCTGGACATCGCCGGTCTGCCCGGC
A.jandaei       CGCAAGGCCCGCGAACTGACCCGCCGCAAAGGCGCGCTGGATATCGCCGGTCTGCCCGGC
A.hydrophi      CGCAAGGCCCGCGAACTGACCCGCCGCAAAGGCGCGCTGGATATCGCCGGTCTGCCCGGC
A.allosacc      CGCAAGGCCCGCGAACTGACTCGCCGCAAAGGCGCGCTGGATATTGCCGGTCTGCCCGGC
A.v.bv._ve      CGCAAAGCTCGCGAACTGACCCGCCGCAAAGGCGCGCTGGATATCGCCGGTCTGCCCGGC
```

```
A.sobria        CGCAAAGCCCGCGAACTGACCCGCCGCAAAGGCGCGCTGGATATCGCCGGTCTGCCCGGC
I.bacteria      CGCAAAGCCCGCGAACTGACCCTCCGCAAAGGCGCGCTGGATATCGCCGGTCTGCCCGGC
A.v.bv._so      CGCAAAGCCCGCGAACTGACCCGCCGCAAAGGCGCGCTGGATATCGCCGGTCTGCCCGGC
                ***** ** ** **  **** *  ***** ** ** ***** ** ***** *********

A.schubert      AAGCTGGCCGACTGCCAGGAAAAAGATCCGGCGCTCTCTGAACTTTACATCGTCGAGGGT
A.eucrenop      AAGCTGGCGGACTGTCAGGAGAAAGACCCGGCCCTGTCCGAACTCTACATAGTGGAAGGG
A.salmonic      AAGCTGGCAGACTGCCAGGAGAAAGACCCGGCCCTCTCCGAACTCTACATAGTGGAAGGG
A.bestiaru      AAGCTGGCAGACTGCCAGGAGAAAGACCCGGCCCTCTCCGAACTCTACATAGTGGAAGGG
A.media         AAGCTGGCTGACTGCCAGGAGAAAGATCCGGCCCTCTCCGAACTCTACATAGTGGAAGGG
A.jandaei       AAGCTGGCTGACTGTCAGGAAAAAGACCCGGCGCTCTCCGAACTCTACATAGTGGAAGGG
A.hydrophi      AAGCTGGCTGACTGTCAGGAAAAAGACCCGGCGCTCTCCGAACTCTACATAGTGGAAGGG
A.allosacc      AAGCTGGCTGACTGTCAGGAAAAAGATCCGGCTCTCTCCGAACTCTACATAGTGGAAGGG
A.v.bv._ve      AAGCTGGCTGACTGTCAGGAAAAAGACCCGGCTCTCTCCGAACTCTACATAGTGGAAGGG
A.sobria        AAGCTGGCTGACTGTCAGGAAAAAGACCCGGCTCTCTCCGAACTCTACATAGTGGAAGGG
I.bacteria      AAGCTGGCTGACTGTCAGGAAAAAGACCCGGCTCTCTCCGAACTCTACATAGTGGAAGGG
A.v.bv._so      AAGCTGGCTGACTGTCAGGAAAAAGACCCGGCTCTCTCCGAACTCTACATAGTGGAAGGG
                ******** ***** ***** ***** ***** ** ** ***** ***** ** ** **

A.schubert      GACTCTGCGGGCGGTTCCGCCAAGCAGGGTCGTAACCGGAAGAACCAGGCCATCCTGCCG
A.eucrenop      GACTCTGCTGGCGGCTCCGCCAAGCAGGGTCGCAACCGCAAGAACCAGGCCATACTGCCA
A.salmonic      GACTCTGCTGGCGGTTCCGCCAAGCAGGGTCGCAACCGGAAGAACCAGGCCATACTGCCG
A.bestiaru      GACTCTGCTGGCGGCTCCGCCAAGCAGGGTCGCAACCGCAAGAACCAGGCCATACTGCCG
A.media         GACTCTGCTGGCGGCTCCGCCAAGCAGGGTCGCAACCGCAAGAACCAGGCTATCCTG-CG
A.jandaei       GACTCTGCTGGCGGTTCCGCCAAGCAGGGTCGCAACCGTAAGAACCAGGCCATCCTGCCG
A.hydrophi      GACTCTGCTGGCGGTTCCGCCAAGCAGGGTCGCAACCGGAANAACCAGGCCATCCTGCCG
A.allosacc      GACTCTGCTGGCGGTTCCGCCAAGCAGGGTCGCAACCGTAAGAACCAGGCTATCCTGCCG
A.v.bv._ve      GACTCTGCTGGCGGTTCCGCCAAGCAGGGTCGCAACCGTAAGGACCAGGCCATCCTGCCG
A.sobria        GACTCTGCTGGCGGTTCCGCCAAGCAGGGTCGCAACCGTAAAAACCAGGCCATCCTGCCG
I.bacteria      GACTCTGCTGGCGGTTCTGCCAAGCAGGGTCGCAATCGTAAGGACCAGGCCATCCTGCCG
A.v.bv._so      GACTCTGCTGGCGGTTCTGCCAAGCAGGGTCGCAATCGTAAGGACCAGGCCATCCTGCCG
                ******** ***** ** ************** ** ** **  ******* ** *** *

A.schubert      CTCAAGGGCAAGATCCTGAACGTGGAGAAGGC----------------------------
A.eucrenop      CTCAAGGGCAAGATCCTGAACGTGGAGAAGGC----------------------------
A.salmonic      CTCAAGGGCAAGATCCTGAA----------------------------------------
A.bestiaru      CTCAAGGGCAAGATCCTGAACGTGGAGAAGGC----------------------------
A.media         CTCAAGGGCA--------------------------------------------------
A.jandaei       CTCAAGGGTAAAATCCTGAACGTGGAGAAGGC----------------------------
A.hydrophi      CTCAAGGGCAAGATCCTGAACGTGGAGAAGGC----------------------------
A.allosacc      CTCAAGGGCAAGATCCTGAACGTAGAGAAGGC----------------------------
A.v.bv._ve      CTCAAGGGTAAAATCCTGAACGTGGAGAAGGC----------------------------
A.sobria        CTCAAGGGTAAAATCCTGAACGTAGAGAAGGCCCGTTTCGACAAGATGATCTCCTCGCAA
I.bacteria      CTCAAGGGTAAAATCCTGAACGTAGAGAAGGC----------------------------
A.v.bv._so      CTCAAGGGTAAAATCCTGAACGTAGAGAAGGC----------------------------
                ******** *

A.schubert      ------
A.eucrenop      ------
A.salmonic      ------
A.bestiaru      ------
A.media         ------
A.jandaei       ------
A.hydrophi      ------
A.allosacc      ------
A.v.bv._ve      ------
A.sobria        GAGGTG
I.bacteria      ------
A.v.bv._so      ------
```

Figure S2: **MS spectrum of the active fraction. (A)** Analysis of the antimicrobial active fractions by MALDI TOF/TOF in positive reflector mode shows two m/z values of 1157.74 and 1298.67. **(B)** Partial identification by fragmentation of the 1151.64 and 1298.67 peaks (left and right respectively). P3 and P4 correspond to degraded products of P1 and P2.

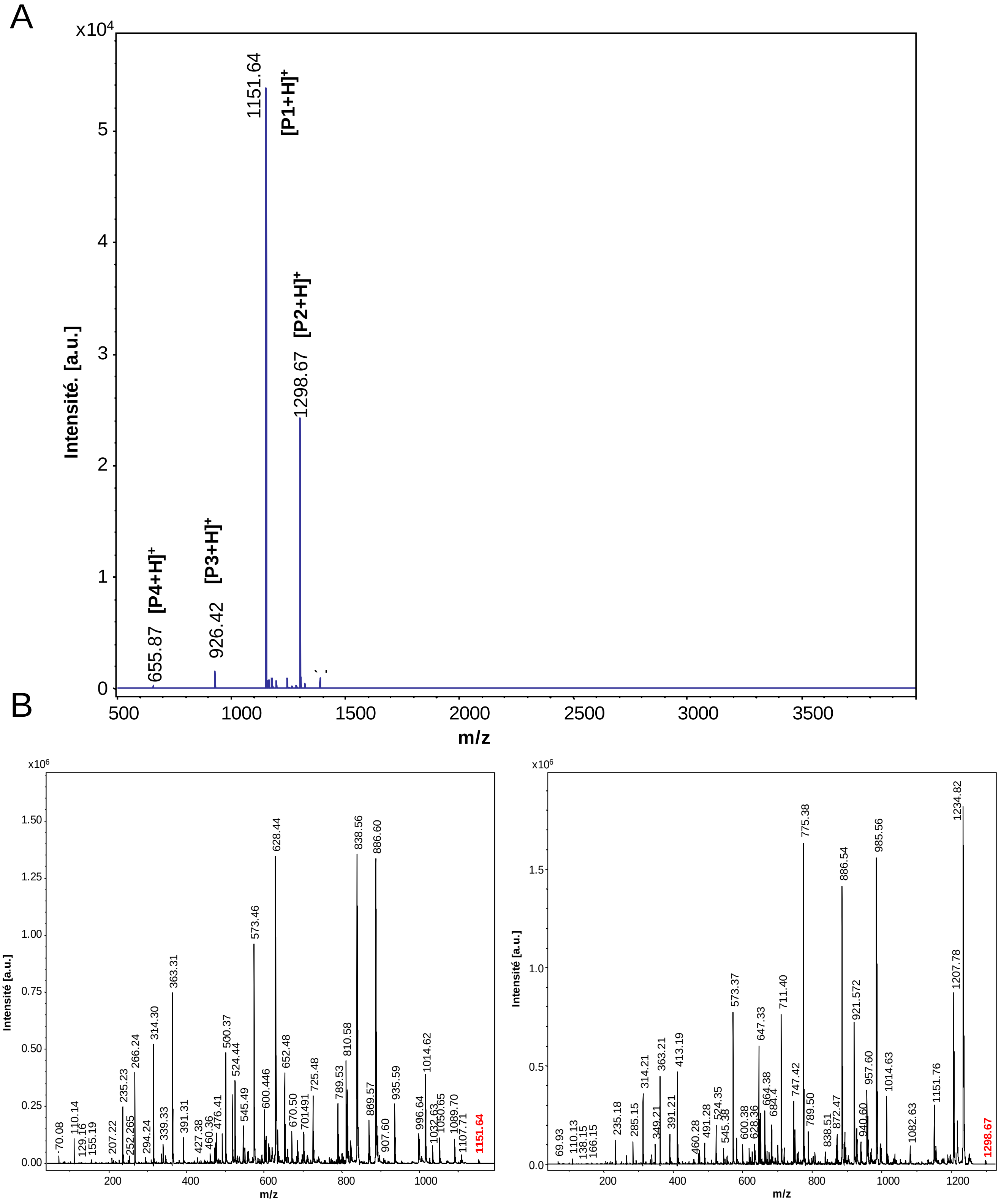

Figure S3: **The anti-TMC antibody cross-reacts with NMC. (A)** ClustalW alignment of the NMC and TMC precursors. The amino acid sequence of TMC recognized by the antibody (anti-TMC epitope) is well conserved between NMC and TMC **(B)** Western blot analysis of entire animals (whole body). Immuno-staining with the anti-TMC Ab revealed two bands of 9 and 10 kDa, corresponding to preNMC and preTMC mass predictions, respectively.

A

```
preNMC   MALLNKLLCFALVFMIFGEFVTPDCYEDWSRCTPGTSFLTGILWKDCHSRCKELGHRG-G 59
preTMC   MELKSG-LSILLCFGICIAVINAGCFEDWSRCSPSTASATGVLWRSCDSYCKVCFKADRG 59
         * * .  *.: * * *   .:...*:******:*.*:  **:**:.*.* **   : . *
preNMC    RCVDSPSKHCPGVLKNNKQCHCY--------------- 82
preTMC    ECYDSPSLNCPHRLPNNKQCRCINARTAKDNRNPTCWA 97
          .* **** :**  * *****:*
```

Anti-TMC epitope

B

Leech
whole body

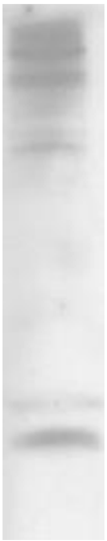

preTMC (10744 Da)
preNMC (9314 Da)

- 15 KDa
- 10 KDa

Figure S4: **TMC and NMC nucleotidic sequences. (A)** ClustalW alignment of the nucleotidic sequences encoding TMC and NMC. The coding sequence (in black) of NMC is shorter than TMC. The TMC/NMC forward primer used for the PCR amplification (highlighted in grey) corresponds to a sequence well conserved between both molecules. **(B)** PCR amplification of cDNA prepared from entire leeches, using the TMC/NMC forward primer and an oligodT revealed two bands corresponding to NMC and TMC.

A

```
Neuromacin      CGTG-ACTCCGGATTGCTACGAGGACTGGAGCAGGTGCACGCCTGGAACGTCATTCCTGA
Theromacin      TGGATAAAAGAGGATGTTTCGAAGATTGGAGTCGTTGTTCGCCATCGACAGCTAGTGCAA
                 *   *     *  ** * *** ** *****  * **  ****    **  *       *

Neuromacin      CTGGAATCCTGTGGAAAGATTGCCACAGCCGTTGCAAGG---AACTCGGTCACAGGGGAG
Theromacin      CAGGAGTTTTATGGAGAAGTTGTGACAGTTACTGCAAAGTTTGTTTCAAAGCTGATAGGG
                * *** *  * **** *  ***  ****    ***** *      **          * *

Neuromacin      GACGATGTGTGGATTCACCAAGCAAACACTGCCCTGGAGTCCTCAAGAACAACAAACAGT
Theromacin      GAGAATGTTATGATTCGCCAAGTCTCAATTGTCCACATCGTCTACCAAATAACAAACAAT
                **  ****   ***** *****     * **  **       **    **  ******** *

Neuromacin      GTCATTGCTACTGATCTGAAGAATTGTTGAAATATGTTGGAGATTCTGGAAGGACAGTGA
Theromacin      GCAGGTGCA--TAAACGCTAGAACTGC-AAAAGACAATAGGAATCC----AACTTGTTGG
                *    ***   * * *   **** **   *** *   * *  ** *    *      **

Neuromacin      CCATGATTTGTGATTTTCTTTAAAAATCACTTAGTTAAGTAAAAACATTGGCGATGATTA
Theromacin      GCTTGATAGGTCGACGGGGAATCACTAGTGAATTCGCGGCCGCCTGCAGGTCGACCATAG
                 * ***

Neuromacin      CGCTTACATAATAAAGTATTATTTTGCGATTGCACCATTCCAAAAAAAAAAAAAAAAAAA
Theromacin      GATGTTTCGAAGATTGGAGTCGTTGTTCGCCATCGACAGCTAGTGCAACAGGAGTTTTAT

Neuromacin      AAA---------------------------------------------------------
Theromacin      GGAGAAGTTGTGACAGTTACTGCAAAGTTTGTTTCAAAGCTGATAGGGGAGAATGTTATG

Neuromacin      ------------------------------------------------------------
Theromacin      ATTCGCCAAGTCTCAATTGTCCACATCGTCTACCAAATAACAAACAATGCAGGTGCATAA

Neuromacin      ------------------------------
Theromacin      ACGCTAGAACTGCAAAAAAAAAAAAAAAAA
```

B

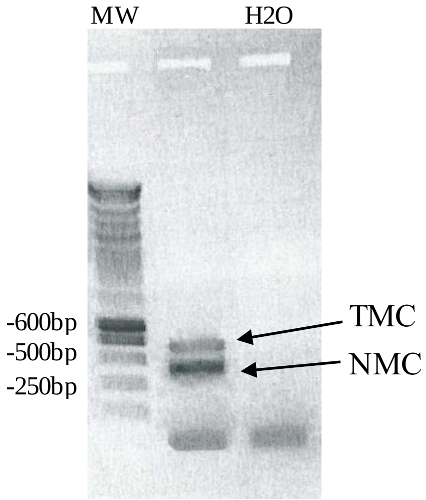

Electronic Supplementary Methods

Antimicrobial assays

Liquid assay: One isolated colony of each bacterial strain to test is cultured in Luria Bertoni (LB) medium at 37°C under agitation (130 rpm) for 18 h. The overnight bacterial cultures is diluted in LB to give 2-7 x 10^5 colony forming units/ml. 100 µl of bacterial suspension are added to 10µl of the sample to test, in a sterile 96-well microtiter plate (CELLSTAR, Greiner bio-one). After 24 h of incubation at 37°C, the OD of each well of the plate is measured at 600nm with a plate reader. The antimicrobial activity appears as an inhibition of the bacterial growth in the well containing an active substance (OD comparable to the culture medium alone).

Solid assay: One isolated colony of each bacterial strain to test is cultured in Mueller Hinton Broth (MHB) medium at 37°C under agitation (130 rpm) for 18h. The overnight culture is then diluted (1:100) in the same culture medium and incubated for 2-3 hours at 37°C under agitation (130 rpm). 230 µl of the bacteria at the DO of 0.01 (Absorbance: 600nm) are mixed to 50 ml of melted MHB-agar. The mix is immediately poured into a petri dish (10 ml /dish). 1 to 4 µl of the substance to test is (are) spotted on the solidified medium containing the bacteria and the plate is incubated overnight at 37°C. Activity appears as a zone of inhibition of the bacterial growth at the deposit site of the tested substance.

Western-blot analysis

3 leeches were crushed with a mortar in liquid nitrogen. The powder was resuspended in lysis buffer (9.5 mM urea, 2% Triton X-100, 65 mM 2-ME, and 1.25% SDS). The protein concentration was determined by the Bradford method using BSA as a standard. SDS-PAGE. The running gel was composed of 10% acrylamide with 150 g of Tris/0.6 N HCl and the stacking gel was composed of 4% acrylamide with 120 g of Tris/0.8 N

HCl. Migration was performed using a cathode buffer (0.6% Tris base, 2.5% taurine, and 0.1% SDS) and an anode buffer (0.6% Tris base, 2.8% glycine, and 0.1% SDS). A total of 50 µg of protein was loaded in Laemmli buffer (120 g of Tris/0.8 N HCl, 50% glycerol, 10% SDS, and 7.7% DTT). Gels were run at 70 V for 15 min and then at 120 V for 45 min. Western blotting. The SDS-PAGE gel was transferred to a nitrocellulose membrane (Hybond-C Extra; Amersham Biosciences) by electro-blotting. After transfer, the membrane was blocked for 1 h in PBS containing 0.05% Tween 20 and 0.7% casein and then probed with the polyclonal anti-TMC Ab (1/1200 dilution) in blocking solution overnight at 4°C. After intensive washes with PBS/0.05% Tween 20, the immune-labeled bands were detected using a peroxidase-conjugated anti-rabbit secondary Ab (1/5000; 90 min at room temperature). An ECL western blotting kit (Amersham Biosciences) was used for chemo-luminescence visualization with Kodak X-Omat AR film.
